# PROTAC vaccine: A new way to live attenuated vaccines

**DOI:** 10.1002/ctm2.1081

**Published:** 2022-10-25

**Authors:** Zhen Li, Haiqing Bai, Xuetong Xi, Wen‐xia Tian, John Z.H. Zhang, Demin Zhou, Longlong Si

**Affiliations:** ^1^ CAS Key Laboratory of Quantitative Engineering Biology Shenzhen Institute of Synthetic Biology Shenzhen Institute of Advanced Technology Chinese Academy of Sciences Shenzhen China; ^2^ Xellar Biosystems Inc Cambridge Massachusetts USA; ^3^ College of Life Sciences University of Chinese Academy of Sciences Beijing China; ^4^ College of Veterinary Medicine Shanxi Agricultural University Jinzhong China; ^5^ State Key Laboratory of Natural and Biomimetic Drugs School of Pharmaceutical Sciences Peking University Beijing China

**Keywords:** influenza, live attenuated vaccine, PROTAC, targeted protein degradation

1

While the SARS‐CoV‐2 pandemic is attracting attention from all over the world, influenza remains a major challenge to global health. Despite the use of existing vaccines, influenza still causes 3–5 million severe cases and .3–.65 million deaths worldwide each year,^1^ highlighting the need for new vaccine strategies that can generate improved vaccines and thus expand our antiviral arsenal. In a proof‐of‐concept study,^2^ Si et al. describe the development of a proteolysis‐targeting chimeric (PROTAC) vaccine technology by using the host cellular ubiquitin‐proteasome system to conditionally degrade influenza viral proteins (Figure 1). The generated PROTAC influenza vaccine is highly attenuated by the host protein degradation machinery and able to elicit robust and broad humoural, mucosal and cellular immune responses against homologous and heterologous viral challenges in mice and ferrets. While this study focuses on the influenza virus, this approach could be extended to the production of live attenuated vaccines against other pathogens.

**FIGURE 1:**
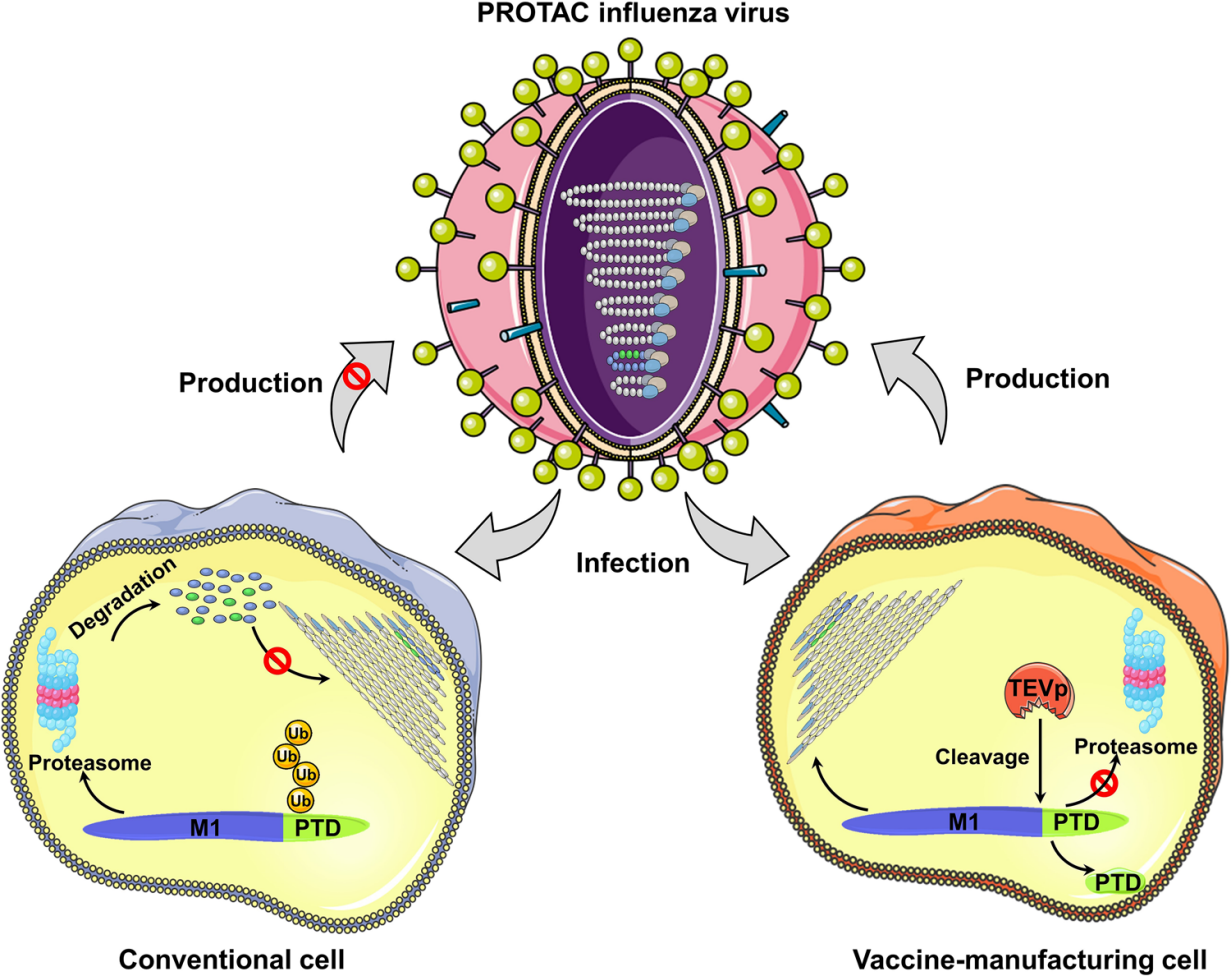
Principle of PROTAC virus vaccine design. PROTAC viruses are attenuated through proteasome‐targeting domain (PTD)‐mediated degradation of influenza viral M1 protein by proteasome in conventional cells (left). PROTAC viruses replicate efficiently for production in cell expressing TEVp that cleave and remove PTD to spare viral M1 protein from degradation. M1, matrix protein; Ub, ubiquitin

The balance between safety and efficacy is a major challenge for vaccine development. Three types of influenza vaccines are currently used clinically: inactivated vaccine, cold‐adapted live attenuated vaccine and recombinant vaccine. While relatively safe, inactivated vaccines provide suboptimal protection against seasonal influenza infections. Attenuated live viruses can often yield highly effective live attenuated vaccines that have the potential to induce robust and broad immune responses.^3^ However, attenuation through adaptive mutations from serial passaging—the commonly used strategy of attenuation—is time‐consuming and cannot guarantee the production of safe viral strains.^3^ For the cold‐adapted live attenuated influenza vaccine FluMist, the attenuating mutations were obtained by lengthy culture and mapped to six viral gene segments, which have to be kept constant every year with only viral hemagglutinin and neuraminidase proteins being from circulating viral strains, thus providing limited antigen match and efficacy.^4^ Recent studies have demonstrated several strategies to design live attenuated vaccines that are in development.^2^, ^4^, ^5^, ^6^, ^7^ Compared to these approaches, the PROTAC vaccine uses a distinct design principle—targeting viral protein to the host's ubiquitin‐proteasome system for degradation and thus attenuating viral replication.

The PROTACs typically refer to chemical small molecules or peptide‐based molecules that contain two covalently linked functional moieties, one specifically binding to a protein of interest (POI) and the other to E3 ubiquitin ligase, and are able to induce ubiquitylation of the POI for its subsequent degradation by proteasome.^8^ By generating PROTAC vaccines, Si et al. expand the application of this targeted protein degradation technology to organisms.

The PROTAC vaccine was engineered to incorporate a conditionally removable proteasome‐targeting domain (PTD) into the M1 protein of the influenza virus. For viral attenuation, the PTD includes a peptide, ALAPYIP, that can be recognized by the von Hippel‐Lindau (VHL) E3 ubiquitin ligase and induce proteasomal degradation of tagged M1 protein in conventional cells (Figure 1). For vaccine manufacture, the PTD includes a tobacco etch virus cleavage site (TEVcs) linker, which can be selectively cleaved by TEV protease (TEVp), separating viral M1 protein from the PTD and thus sparing M1 protein from degradation in engineered TEVp‐expressing cells (Figure 1).

As expected, the PTD mediates viral M1 protein degradation and attenuation of the resultant PROTAC vaccine strain in proteasome‐ and VHL E3 ubiquitin ligase‐dependent manner. The PROTAC vaccine strain efficiently replicated in TEVp‐expressing Madin‐Darby canine kidney (MDCK.2) cells, which will potentially enable large‐scale production of the PROTAC vaccine. In contrast, the replication competence of the PROTAC vaccine strain decreased by > 2×10^4^‐fold relative to wild‐type (WT) virus in conventional MDCK.2 cells. In vivo attenuation was observed in mice and ferrets: the replication competence of the PROTAC vaccine strain decreased by 10^4^‐ to 10^4.6^‐fold relative to the WT virus in mice and by 10^2^‐ to 10^2.9^‐fold in ferrets. The authors further show that the PROTAC vaccine strain was able to elicit robust and broad immunity, including humoural, mucosal and cellular immune responses, and provided cross‐reactive protection against homologous and heterologous influenza viral challenges in mice and ferrets. Furthermore, the authors demonstrate that PTD‐mediated viral protein degradation by the proteasome enhanced presentation of viral antigens,^2^ which is critical for inducing strong T cell responses.^9^

Despite these promising preclinical results, further investigations are needed before applying this approach to human use. In this proof‐of‐concept study, the VHL E3 ubiquitin ligase was chosen to construct the PROTAC vaccine. However, potential loss or dysfunction of VHL in some patients with VHL diseases^10^ raise potential safety concerns for this PROTAC vaccine strain in these patients. Fortunately, more than 600 E3 ubiquitin ligases have been found^2^, ^11^ in human, and numerous PTDs recognized by other E3 ubiquitin ligases can be designed and used to generate diverse PROTAC vaccine strains, which could enable personalized selection for populations with different genetic backgrounds, such as those with defective expression of a specific E3 ubiquitin ligase. In addition, PROTAC vaccines may not suit those patients who are receiving therapy with proteasome inhibitors, such as Bortezomib.

To generate PROTAC vaccine strains, the authors conducted a screening by incorporating the PTD to each of the eight viral proteins (M1, PB2, PB1, PA, NP, M2, NEP and NS1). Only one strain (M1‐PTD) was successfully generated when the PTD was incorporated into the viral M1 protein. As the authors purposed, the conformations of PTD within its tagged viral proteins could be partially responsible for the failure in generating other PROTAC vaccine strains: the VHL‐binding peptide is exposed but the TEV cleavage site linker is buried in these seven viral proteins, leading to efficient proteolysis but preventing cleavage required for vaccine production. Therefore, although this PROTAC vaccine technology could be applicable to other pathogens, choosing suitable viral protein(s) will be critical: (1) the incorporation of PTD to viral proteins should not significantly affect the structures and functions of viral proteins; (2) the incorporated PTD needs reasonable conformations that enable efficient E3 binding for proteasome degradation and efficient cleavage to remove PTD for large‐scale production. Future research is needed to better understand the structural basis of these factors in order to increase the success rate of new PROTAC vaccine designs.

## CONFLICT OF INTEREST

The authors declare no conflict of interest.
